# Genetic mapping of *shn^E.3.2^* in *Drosophila melanogaster*

**DOI:** 10.17912/micropub.biology.000118

**Published:** 2019-06-05

**Authors:** Kayla Bieser, Jamie Siders Sanford, Ken Saville, Katherine F. Arreola, Zachary T. Ayres, David Basulto, Serena Benito, Christopher J. Breen, Julian A. Brix, Nicole Brown, Krissa K. Burton, Taree M. Chadwick, Matthew Chen, Katherine Chu, Beverly L. Corbett, Zerrick Dill, Meghan A. Faughender, Ashlynn D. Hickey, Joshua S. Julia, Shannon S. Kelty, Briggette B.K. Jobs, Bryce A. Krason, Brian Lam, Colin L. McCullough, Bryanna R. McEwen, Julian L. McKenzie, Kayla R. McQuinn, Chloe M. Moritz, Kristina E. Myers, Elizabeth M. Naugle, Ashley M. Nutter, Danielle Q. O'Conke, Megan T. O'Grondik, Kriya B. Patel, Sydney M. Rudowski, Emma N. Sberna, Gunner M. Stall, Tad L. Steiner, Eda Tanriverdi, Natalie Torres Patarroyo, Virginia L. Traster, Leo P. Tsai, Andrew J. Valenti, Mariela M. Villegas, Samantha M. Voors, Kierra K. Watson, Megan E. Wright, Jacob D. Kagey

**Affiliations:** 1 Department of Physical and Life Sciences, Nevada State College; 2 Department of Biological and Allied Health Sciences, Ohio Northern University; 3 Department of Biology, Albion College; 4 Biology Department, University of Detroit Mercy

**Figure 1.**
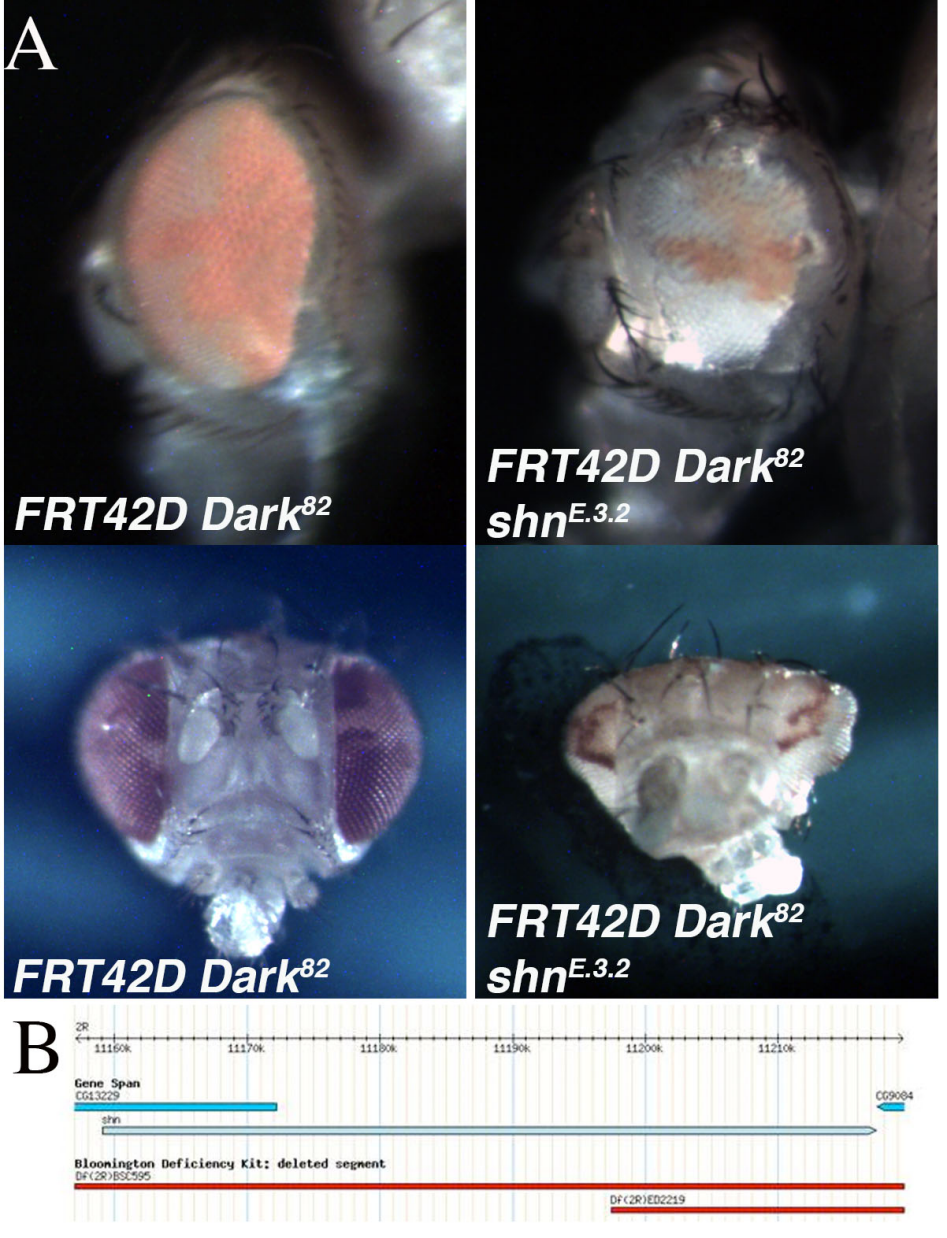
A. Mosaic control (*FRT42D Dark^82^*) and mosaic mutant E.3.2 (*FRT42D Dark^82^shn^E.3.2^*) eyes. In both genotypes, homozygous mutant eye tissue is pigmented (*w^+mC^*). The top panels are representative images of the lateral view and bottom panels the medial view. B. Genomic region of chromosome 2R in which mutant E.3.2 failed to complement with deficiency mapping (2R:11,186,047..11,665,391). The *shn* gene falls within this region. B. Image adapted from flybase.org (Gramates et al., 2017).

## Description

An EMS screen was conducted utilizing the Flp/FRT system to identify mutations that lead to phenotypic alterations in the size of the eye, the ratio of mutant to wild type tissue (red over white), or the developmental patterning of the mosaic eye. This screen was completed in the genetic background of blocked apoptosis in the homozygous mutant cells to identify conditional regulators of cell growth and eye development (Kagey *et al.,* 2012). The block in apoptosis in the mosaic mutant tissue was achieved by using the *FRT42D Dark^82^*chromosome, which retains the *w^+mC^*(pigmentation), as a starting point for the EMS mutagenesis (Akdemir *et al.,* 2006). One of the mutants identified was mutant E.3.2. The mutant mosaic phenotype, generated from the cross *FRT42D Dark^82 ^E.3.2* X*Ey>Flp; FRT42D*, resulted in a range of phenotypes. This included, gross eye pattern disruption, abnormal shape, and antennal overgrowth (see bottom image) when compared to the *FRT42D Dark^82 ^*mosaic controls (Figure 1A). The control mosaic phenotype had a characteristic 60:40 red:white ratio as compared to the mutant mosaic phenotype of approximately 30:70 red:white ratio. This indicates a reduction in mutant tissue, but included abnormal cranial, eye, and antennal development (Figure 1A).

The genetic mapping of the location of *E.3.2* on 2R was completed by three independent groups of undergraduate researchers at Nevada State College, Albion College, and Ohio Northern University as part of the Fly-CURE consortium (Bieser *et al.,* 2018, Stamm *et al.,* 2019). Virgin females from the *FRT42D E.3.2 Dark^82^/CyO* stock were mated in series to male flies from the 87 deficiency stocks that comprise the Bloomington Stock Center 2R Deficiency Kit (only stocks distal to the FRT42D site were used for mapping) (Cook *et al.,* 2012). Mutant *E.3.2* failed to complement deficiencies *Df(2R)BSC595* (2R:10,385,967..11,288,578), *Df(2R)ED2155* (2R:10,894,096..11,397,442), and *Df(2R)ED2219* (2R:11,197,412..11,665,391), while complementing the flanking deficiency *Df(2R)Exel6059* (2R:10,874,385..11,186,047). This resulted in the genomic region of 2R:11,186,047..11,665,391 failing to complement (Figure 1B). A lethal allele for the candidate gene *Schnurri,* *shn[1]*,was crossed independently to *E.3.2* to test for complementation. E.3.2 failed to complement a previously defined strong hypomorphic allele, *shn[1]* (Horsfield *et al.,* 1998)*,* indicating that *E.3.2* is a novel *shn* allele, *shn^E.3.2^*. *Shn* is a zinc-finger transcription factor known to act in complex with Mothers Against Dpp (mad) in response to the BMP-related Decapentaplegic (*Dpp*) signaling pathway (Dai *et al.,* 2000; Udagawa *et al.,* 2000). *Dpp*, a member of the TGFβ superfamily, is a *Drosophila* morphogen critical for directing early embryonic patterning and regulating cell growth. *Dpp* mutants have shown that *Dpp* signaling is necessary for proper patterning in the eye-antenna disc in the L2 and L3 wandering larval stages of Drosophila development (Won *et al.,* 2015). The current work is in agreement with previous findings and indicate that perturbations of *shn*, a downstream target of *Dpp*, is involved in regulation of cell growth and developmental patterning *in vivo*.

## Reagents

*FRT42D Dark^82^/CyO* (Akdemir *et al.,* 2006)

*FRT42D Dark^82^ shn^E.3.2^/CyO* (this manuscript)

*Ey>Flp; FRT42D* (BDSC 5616)

Bloomington Drosophila Stock Center 2R Deficiency Kit (Cook *et al.,* 2012)

*w^1118^;Df(2R)BSC595/CyO* (BDSC 25428)

*w^1118^;Df(2R)ED2219,P{w[+mW.Scer\FRT.hs3]=3’.RS5+3.3’}ED2219/Sm6a* (BDSC 8910)

*w^1118^;Df(2R)ED2155,P{w[+mW.Scer\FRT.hs3]=3’.RS5+3.3’}ED2155/SM6a* (BDSC 9344)

*w^1118^;Df(2R)Exel6059,P{w[+mC]=XP-U}Exel6059/CyO* (BDSC 7541)

*cn[1] shn[1] bw[1] sp[1]/CyO* (BDSC 3008)
